# Square-stepping Exercise for Older Adults Attending a Daycare Service Facility: A Feasibility Study

**DOI:** 10.1298/ptr.25-E10387

**Published:** 2026-04-29

**Authors:** Ryota UCHIDA, Takashi KUROSAKI, Ryosuke SHIGEMATSU, Hiroyuki SASAI, Yuto HIKIJI, Jessica KOSCHATE-STORM, Svenja TIETGEN, Masaki NAKAGAICHI

**Affiliations:** 1 Health and Sports Promotion Department, Sports Innovation Organization, National Institute of Fitness and Sports in KANOYA, Japan; 2 Minamiosumi Town Hall, Japan; 3 School of Health and Sport Sciences, Chukyo University, Japan; 4 Research Team for Promoting Independence and Mental Health, Tokyo Metropolitan Institute for Geriatrics and Gerontology, Japan; 5 Master’s Course in Physical Education, National Institute of Fitness and Sports in KANOYA, Japan; 6 Department of Health Services Research, Faculty VI Medicine and Health Sciences, Carl von Ossietzky Universität Oldenburg, Germany; 7 Department of Geriatrics, General Hospital Bremerhaven Reinkenheide gGmbH, Germany; 8 Sports and Life Sciences, National Institute of Fitness and Sports in KANOYA, Japan

**Keywords:** Recreational activity, Safety, Adherence, Acceptability

## Abstract

**Objectives:**

Square-stepping exercise (SSE) is a preventive program that reduces the risk of falls and improves physical function and agility in community-dwelling older adults. Although SSE has shown benefits in community-dwelling older adults, its feasibility in Japanese daycare service facilities, where physical and cognitive conditions may differ, remains unclear. This study aimed to assess its implementation and acceptability among older adults in daycare service facilities.

**Methods:**

A single-arm, 4-week intervention was conducted at Daycare Service Facility A in Kagoshima, Japan. Participants performed SSE once weekly for 4 weeks, with each session lasting 60 min. Safety (absence of injuries or illnesses), program completion rates, and session attendance rates were monitored throughout the intervention, and one-on-one semi-structured interviews were conducted to assess acceptability.

**Results:**

The participants were 21 older adults (mean age 87.4 ± 4.6 years). Adherence was high, with a 95.2% completion rate and 100% attendance rate. No injuries were observed. All participants completed the exercises by following the step patterns, as instructed. Interviews revealed positive psychological changes, such as enjoyment and a sense of accomplishment, and physical improvements, including better lower-limb conditions and overall health.

**Conclusions:**

The findings suggest that SSE may be safely implemented with high adherence in older females with care levels of 3 or below in daycare service facilities and may also promote positive psychological changes. It is necessary to conduct a more detailed evaluation of its effectiveness.

## Introduction

In Japan, the number of individuals using Japan’s Long-Term Care Insurance system has continued to increase due to the country’s aging population. It rose from 6593000 in April 2019 to 7101000 in April 2024^1)^. As various medical and long-term care challenges are expected to intensify, such as rising social security costs and a shortage of caregivers, it is essential to further promote efforts toward long-term care prevention.

According to the Japanese Ministry of Health, Labour and Welfare^2)^, care prevention is defined as “preventing (delaying) the occurrence of a condition requiring long-term care as much as possible, and even if a person is in a condition requiring long-term care, preventing its deterioration as much as possible and ideally reducing it.” In other words, it is important to both prevent the onset of long-term care needs and to mitigate their severity.

To address the progression of care needs, daycare service facilities in Japan provide support such as bathing and meal assistance, along with recreational activities and functional training for individuals who require care. To maintain and improve physical function while reducing the burden on facility staff, there is a growing need to implement effective and efficient functional training programs.

Square-stepping exercise (SSE)^3)^ is a preventive exercise program for older adults. It is performed on a mat that is 100 cm wide and 250 cm long and divided into 25 cm squares. SSE has been reported to reduce the risk of falls and improve physical function and agility in older adults^3,4)^.

In a recent study, the SSE has been considered for use in older adults with reduced physical function. SSE has improved physical function in patients admitted to acute geriatric wards^5)^. SSE is a group-based program that incorporates walking movements; however, because participants take turns stepping one at a time, it has the potential to enhance safety while reducing the burden on facility staff. If the SSE can be implemented as part of the functional training offered at daycare service facilities, it may contribute to the prevention of further physical decline in individuals receiving long-term care.

It has been emphasized that a feasibility study should be conducted to confirm the viability of the intervention prior to the main study^6)^. Evaluation is expected to include not only quantitative measures but also qualitative data, such as interview results, which might help ascertain the acceptability of an intervention, willingness to take part, or ways to refine particular components of the intervention^7)^. Pilot studies, which are a type of feasibility study, are commonly conducted when introducing new exercise programs for older adults to evaluate feasibility and acceptability^8,9)^. Furthermore, systematic reviews and meta-analyses have reported that adherence rates to combined exercise programs, including balance and resistance training, remain high, reaching approximately 85% even among older adults with sarcopenia^10)^.

This study aimed to examine the feasibility of the SSE in older adults who use daycare services. Moreover, particular emphasis was placed on the individuality of acceptability, aiming to qualitatively identify facilitators and barriers that might have been overlooked by quantitative indicators. The study focused on 2 key aspects: implementation and acceptability, with reference to the framework proposed by Bowen et al.^6)^. We evaluated whether SSE could be implemented safely and with high completion and attendance rates, as well as participants’ impressions and acceptability of SSE.

## Methods

### Study design

This was a single-arm, 4-week interventional study conducted at Daycare Service Facility A in Tarumizu, Kagoshima, Japan, between May and July 2023. It was conducted in accordance with the Declaration of Helsinki. The research protocol was submitted to and approved by the Ethics Review Subcommittee of the National Institute of Fitness and Sports in Kanoya (approval number: 23-1-3). All the participants provided written informed consent.

### Participants

Participants were all clients of Daycare Service Facility A. They were community-dwelling older adults who typically used the facilities every Wednesday or Thursday. The exclusion criteria were as follows: (1) individuals unable to provide consent; (2) those certified as needing long-term care levels 4 or 5, as they were expected to have difficulty performing exercises in a standing position; (3) those with a care level of 3 or below who were unable to walk 10 m continuously, with or without a walker, as they were considered unable to step across the SSE mat and return to the starting position; and (4) individuals with cognitive conditions that prevented understanding or execution of the SSE program. However, those with mild cognitive decline who were able to perform SSE in accordance with the safety precautions described hereafter, as assessed by the SSE instructor, were not excluded.

Japan’s Long-Term Care Insurance system is a public scheme designed to support older adults who need assistance with daily living because of physical or cognitive declines. Applicants are classified into 7 levels based on physical and cognitive function assessments: support levels 1 and 2 (requiring some assistance) and care levels 1–5 (requiring increasing levels of care). The levels of available services vary accordingly.

Certification is based on municipal and medical evaluations, supported by computerized care-hour calculations. For example, a person certified as care level 3 typically requires assistance in all aspects of daily living^11)^.

### Intervention

The SSE includes various step patterns based on the participants’ skill levels^3)^. In this study, 5 elementary step patterns were selected and introduced sequentially, beginning with the least physically demanding task (Fig. 1).

**Fig. 1.**
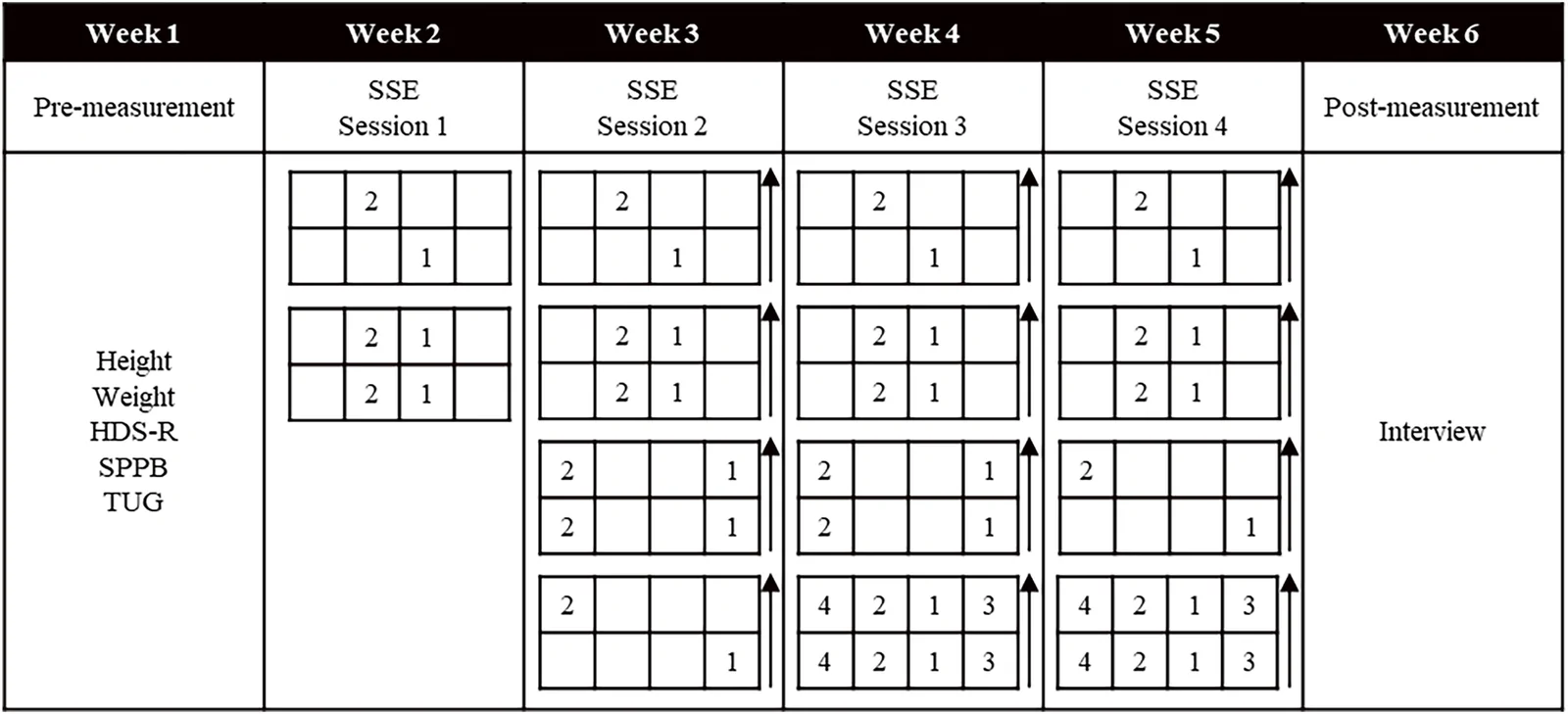
Intervention and measurement protocols. “↑” indicates the direction of the steps, and the numbers in the squares indicate the order of the steps. Odd and even numbers represent the right and left feet, respectively. SSE, square-stepping exercise; HDS-R, Hasegawa Dementia Scale-Revised; SPPB, Short Physical Performance Battery; TUG, Timed Up and Go Test

The intervention period (4 weeks) and frequency (once per week) were determined in consultation with daycare service facility staff. These parameters were chosen based on scheduling constraints due to other annual programs and the SSE instructor’s prior experience, which indicated that 4 sessions would be sufficient to test all 5 elementary step patterns (Fig. 1).

At Daycare Service Facility A, the 60-min period from 14:00 to 15:00 h is usually recreation time. The time usually dedicated to cultural activities and upper-limb exercises in a seated position was fully allocated to the SSE program during the intervention period. Each 60-min session consisted of gathering (10 min), preparation (5 min), SSE (30 min), cooldown (5 min), and dismissal (10 min).

The sessions were held in group-based format: 1 group (11 participants) on Wednesdays and another (10 participants) on Thursdays. One SSE instructor and 2 assistants (daycare service facility staff members) were assigned to each group (Fig. 2). The interventional content was standardized across both groups.

**Fig. 2.**
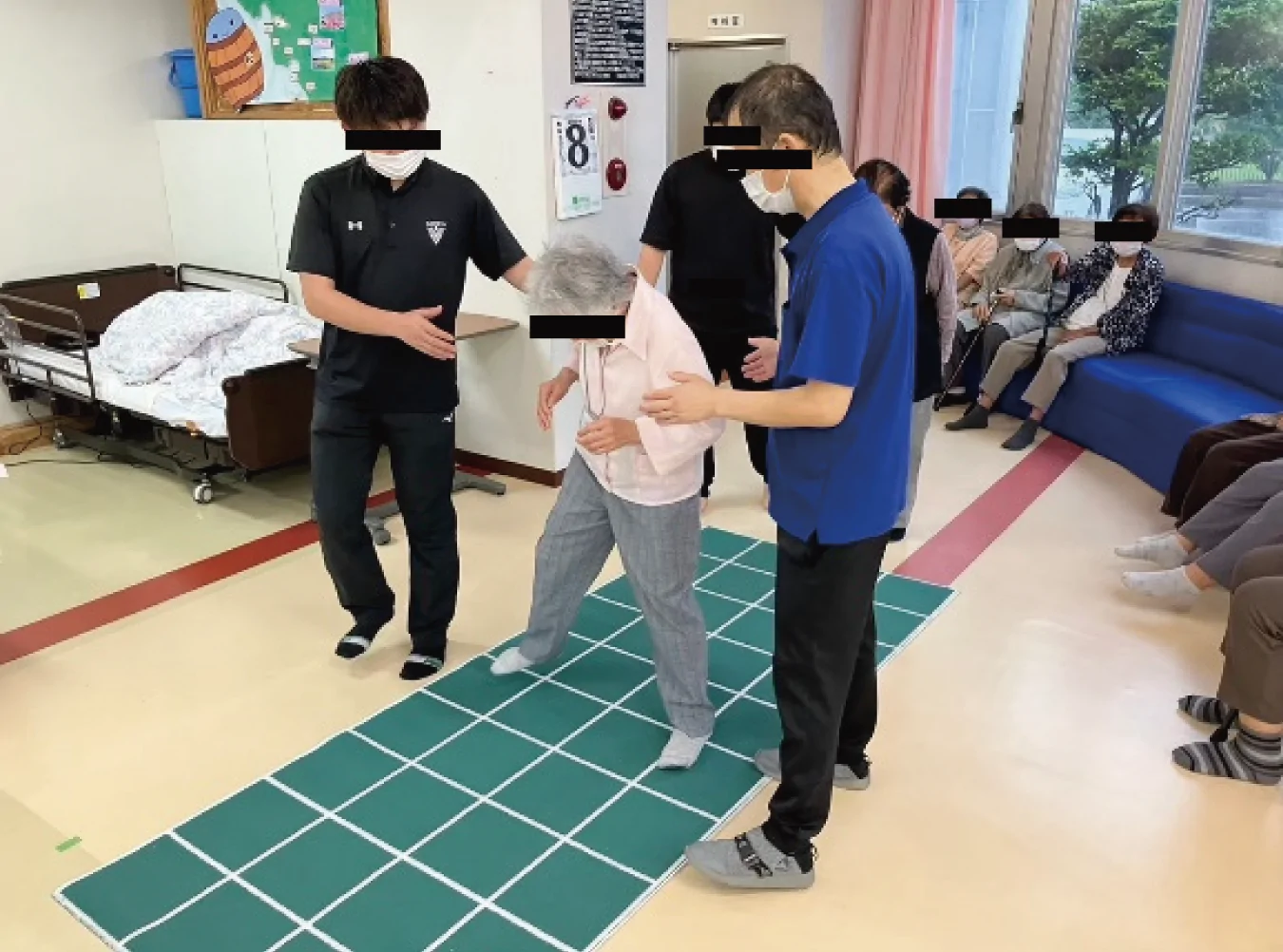
Square-stepping exercise at the facility.

Participants were instructed to follow 2 safety precautions during SSE: (1) move in only 1 direction on the SSE mat and (2) begin stepping only after confirming the instructor’s cue. Before each step, the instructor demonstrated the pattern, and participants performed the steps individually after observing the demonstration.

### Measurements

Bowen et al.^6)^ proposed that feasibility studies should address 8 general areas of focus: acceptability, demand, implementation, practicality, adaptation, integration, expansion, and limited efficacy testing. Based on this report, the primary outcome of this study was the implementation and acceptability of SSE.

### Basic characteristics

Participants’ ages were recorded, and their height and weight were measured. Height was recorded in 0.1-cm increments using a stadiometer. Weight was recorded in 0.1-kg increments using a dual-frequency body composition analyzer (DC-430A-N; TANITA, Tokyo, Japan), with a fixed deduction of 0.5 kg for clothing. Body mass index was calculated as weight (kg) divided by height squared (m²).

### Implementation

Implementation was evaluated from 3 aspects: participants’ function (cognitive and physical), safety, and adherence.

Cognitive function was assessed using the Hasegawa Dementia Scale-Revised (HDS-R)^12)^, administered by a nurse at the facility during a face-to-face interview.

To assess physical function, the Short Physical Performance Battery (SPPB) and the Timed Up and Go (TUG) test were used. A licensed physical therapist performed the assessments. The SPPB evaluates lower-limb function using 3 components: balance, gait speed, and chair stands. Each item was scored on a 4-point scale, with a maximum total score of 12, as described by Guralnik et al.^13)^. Balance tests were conducted in the following sequence: feet together, in semi-tandem, and tandem. The participants progressed to the next test if they could maintain their balance for 10 s. Gait speed was measured over a 4-m distance at the participant’s usual walking pace, and the fastest of the 2 trials was recorded. For the chair stand test, the participants rose from a 45-cm chair 5 times as quickly as possible, and the total time was recorded once. The TUG test was performed twice, measuring the time from when the participant initiated the movement to when they returned to the seated position. The fastest time was used. The participants were instructed to walk with maximal effort, but safely.

To evaluate the safety of the SSE, the presence or absence of injuries or illnesses during or after each session was recorded. The intervention was considered safe if no adverse events were reported.

Adherence was assessed using the following indicators based on prior studies^14,15)^: (1) program completion rate (percentage of participants who completed all sessions) and (2) session attendance rate (percentage of total sessions attended). Following Rivera-Torres et al.^16)^ and Picorelli et al.^15)^, a completion rate of at least 86% and an attendance rate of at least 75% (i.e., attendance at 3 or more sessions) were considered indicative of high adherence. Additionally, the ability to (3) complete the step patterns as instructed and (4) follow safety precautions (i.e., moving in 1 direction and stepping only after the cue) was recorded.

### Acceptability

A one-on-one semi-structured interview was conducted after the intervention to explore the acceptability of SSE (participants’ impressions and subjective effects of the SSE). The interviews were recorded using a digital voice recorder. An independent interviewer, not involved in the SSE sessions, conducted the interviews using the following questions:

(i) Impressions of the SSE (“How did you experience the SSE program?” “What did you think about the SSE?” “Please share your preferences, motivation, and any concerns you had about SSE,” and “Did anything about the training sessions feel stressful?”)

(ii) Subjective effects of the SSE (“Did you feel any benefits from the SSE program?”)

### Data analyses

Baseline cognitive and physical function scores were calculated as the mean, standard deviation (SD), maximum, and minimum values. Program completion rate and session attendance rate were calculated. The interview data were analyzed using qualitative content analysis^17)^. Verbatim transcripts were created from the interviewers’ recordings. The contextual segment of each response was the unit of analysis. A coding frame was constructed using a data-driven approach^18)^. The statements were abstracted and grouped into subcategories based on similarity, and main categories were developed to encompass these subcategories. Interview data were analyzed using NVivo (version 15, Lumivero, LLC, Denver, Colorado, United States of America).

## Results

During the study period, 77 individuals used Daycare Service Facility A. Among them, 65 used the facility on Wednesdays (n = 31), Thursdays (n = 33), or both days (n = 1). Exclusion criteria were applied as follows: individuals who did not provide informed consent (n = 16), those requiring long-term care at levels 4 or 5 (n = 18), and those certified at care level 3 or below but unable to walk 10 m continuously with or without a walker (n = 10). After applying these criteria, 21 participants were included in the SSE intervention (mean age: 87.4 years, SD = 4.6; 1 male and 20 females; 11 Wednesday clients and 10 Thursday clients). Regarding long-term care levels, 8 participants were classified as support level 1, 3 as support level 2, 9 as care level 1, and 1 as care level 3. None of the participants had any previous SSE experience (Fig. 3).

**Fig. 3.**
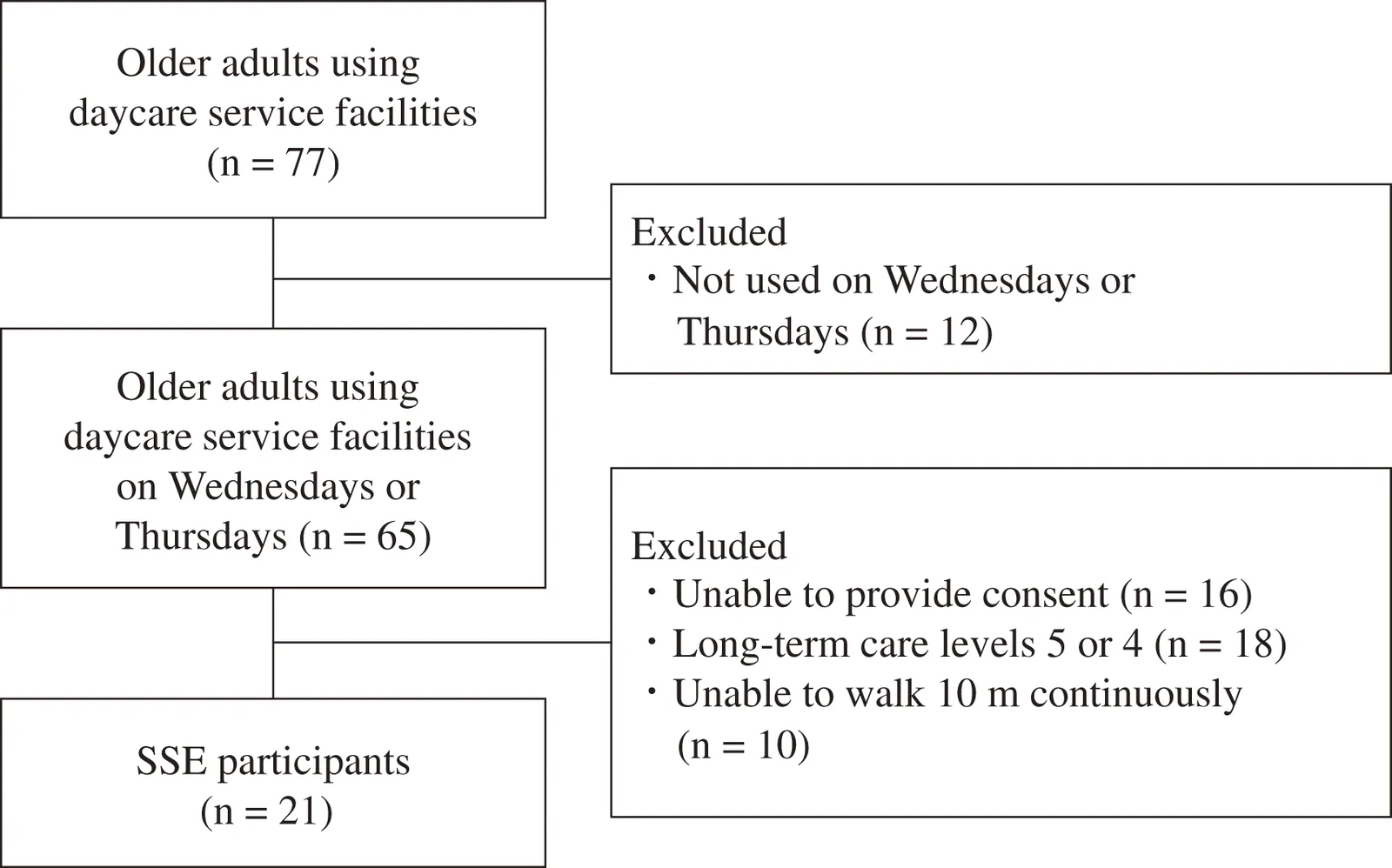
Flowchart of participant decisions. SSE, square-stepping exercise

The baseline cognitive and physical function scores were as follows: the mean HDS-R score was 21.3 (SD = 3.7), ranging from 16 to 30 points. The mean SPPB score was 7.6 (SD = 3.2), ranging from 2 to 12. The mean TUG result was 15.4 s (SD = 7.5), with individual scores ranging from 8.2 to 36.6 s (see Table 1).

**Table 1.** Baseline characteristics and cognitive and physical functions

Age (years)	87.4 ± 4.6
Gender (male/female)	1/20
Height (cm)	145.9 ± 4.7
Weight (kg)	48.6 ± 9.5
BMI (kg/m^2^)	22.8 ± 4.2
HDS-R score (points)	21.2 ± 3.7
SPPB total score (points)	7.6 ± 3.2
TUG (s)	15.4 ± 7.5

Value: mean ± standard deviation.

BMI, body mass index; HDS-R, Hasegawa Dementia Scale-Revised; SPPB, Short Physical Performance Battery; TUG, Timed Up and Go Test

Of the 21 participants, 1 male did not attend sessions 2–4, citing a lack of motivation to continue exercising. Thus, the program completion rate was 95.2%. The remaining 20 participants attended all 4 sessions, resulting in a 100% session attendance rate among the completers. Each participant performed the SSE for 30 min per session. Over 4 weeks, 8 step trials were conducted, including repetitions of 2 different step patterns during week 2. All participants completed each step pattern as instructed. In addition, all participants followed the specified safety precautions during SSE. No intervention-related injuries or health problems were reported during the study period. During the SSE, several participants nearly lost their balance; however, with timely support from instructors or assistants, no falls occurred.

Participants were observed clapping and cheering for one another during the SSE. Their active participation was also observed, as they exchanged positive comments with SSE instructors and other participants.

Tables 2 and 3 provide the qualitative results of the post-intervention interviews. Participants’ impressions of the SSE included both positive and negative psychological reactions. Positive responses included enjoyment and a sense of accomplishment, whereas negative reactions included feelings of difficulty or anxiety. A few participants reported negative physical sensations, such as concerns about falling, reduced physical function, or leg pain. No negative responses were received from the 1 male participant who did not attend sessions 2–4. Regarding the perceived effects of SSE, participants reported positive physical changes such as improved lower-limb conditions and overall health, along with psychological changes such as increased calmness and confidence.

**Table 2.** Feedback on square-stepping exercise

Main category name	Subcategory name	Description of the meaning of the subcategory name	Example of statements
Physical aspect	Concerns about falling	Negative statements about falling	“I felt my right leg got tripped (by the SSE mat).”
	Reduced physical function	Negative statements regarding reduced physical function	“When I say about stress, it would be on me. Being unable to raise my right leg made me stress.”
	Leg pain	Negative statements about physical pain after SSE	“My right leg hurts.”
Psychological aspect	Enjoyment	Positive statements about the Enjoyment of SSE	“It was fun.”
	Sense of accomplishment	Positive statements about the accomplishment	“I thought I couldn't do it, but I finally could.”
	Difficulty	Negative statements about the difficulty of SSE	“It is not easy for an old person like me to do what I want.”
	Anxiety	Negative statements regarding anxiety about stepping correctly	“I just wonder if I can do well as the instructor advises.”

“How did you experience the SSE program?” “What did you perceive about SSE?” “I would like to know your preference, motivation, and concerns about SSE.” “Did you experience anything stressful during the training sessions?”

SSE, square-stepping exercise

**Table 3.** Feedback on square-stepping exercise

Main category name	Subcategory name	Description of the meaning of the subcategory name	Example of statements
Physical aspect	Lower-limb conditions	Positive statements about lower-limb conditions. “Lighter legs,” "Legs lifted,” “Walking better,” “Relief of pain”	“I feel a little lighter on my legs. I had used a time to get my pants on. I've been able to put on pants more easily recently.”
	Overall health	Positive statements regarding improvement in overall health. “Feel good,” “Feel light,” “Move well”	“It makes me feel physically better.”
Psychological aspect	Calmness	Positive statements about psychological aspects. “Calming,” “Feel good,” “Lighthearted”	“I laughed with everyone and became calmer.”
	Confidence	Positive statements regarding increased confidence	“It now seems that I can do what I couldn't do before.”

“Did you get any effects from the SSE program?”

SSE, square-stepping exercise

## Discussion

This study implemented an SSE program during recreation time at a daycare service facility to examine its feasibility for older adults who use daycare services. The study aimed to determine whether SSE can be performed safely and with high adherence. Additionally, participant interviews were conducted to explore their impressions of the program and its perceived effects.

No injuries or illnesses occurred during the study period. The program completion rate was 95.2%, and the session attendance rate among those who completed the program was 100%. Although the number of intervention sessions was low, these rates were higher than those reported for other exercise programs in previous studies^10)^. Additionally, all participants completed the assigned step patterns as instructed and adhered to safety protocols. Even participants with an SPPB score as low as 2 points or a TUG result of 36.6 s could complete the program safely, supported by the SSE instructor and additional staff members. These findings suggest that the SSE can be applied even in older adults with reduced physical function, provided that they can maintain a standing position and complete the TUG test. The use of low-difficulty step patterns and adequate staff support, including 3 staff members per session, is an important consideration for safe implementation.

Participants’ impressions of the program revealed several positive psychological responses, such as enjoyment and a sense of accomplishment. These emotions were evident in their active engagement, including clapping, cheering for one another, and exchanging positive comments with instructors and fellow participants. Such interactions may enhance social connectedness and reinforce positive emotions. Previous research has shown that older adults emphasize fun and enjoyment of social interaction as a motivation to be physically active^19)^. Moreover, the perceived effects of SSE included improvements in physical condition, particularly in the lower limbs, and psychological changes such as increased calmness and confidence. These outcomes may stem from the characteristics of the SSE, which involves a standing movement and allows participants to experience a sense of achievement by completing the step patterns. Awareness of the physical and psychological benefits of exercise is known to encourage ongoing participation^20)^. Therefore, the positive sensations associated with SSE are likely to support continued engagement in the program in the future.

However, some participants reported negative psychological and physical reactions to the SSE program. These included feelings of difficulty and anxiety, concerns about falling, reduced physical function, and leg pain. Compared to regular walking, SSE involves continuous multidirectional movements and requires participants to accurately follow step patterns, presenting a dual-task challenge that engages both cognitive and physical functions. For example, 1 female participant with an HDS-R score of 16 described the SSE as “difficult,” and another female participant with an SPPB score of 2 expressed dissatisfaction related to reduced physical function. These observations suggest that the cognitive and physical function may have influenced participants’ psychological and physical reactions to the SSE program. These characteristics may have contributed to the emergence of such negative reactions, which is 1 limitation of the SSE. To minimize these psychological and physical adverse effects, SSE instructors may need to refine their communication strategies and provide appropriate physical support during the sessions.

This study has several limitations that should be considered when interpreting the findings. First, this study included only 1 male participant, who did not attend sessions 2–4 due to a lack of motivation. Although no negative responses toward the SSE were reported by this participant, the reasons for his absence could not be explored in detail. Consequently, the feasibility and acceptability of SSE in older males could not be evaluated. Previous research on adults in their 60s has reported that, as a motivation for physical activity, females tend to value sharing time with others and socializing with friends, whereas males prefer activities that can be performed alone^21)^. Given that SSE is conducted in a group setting and involves interaction with others, its program characteristics may reduce acceptability among males. Future studies should aim to include a more gender-balanced sample to explore potential differences in program feasibility and acceptability. Second, as the present findings are based solely on participants who accepted the intervention, implementation, and acceptability may have been overestimated, and the generalizability of the results is limited. It should also be noted that these findings primarily reflect the feasibility and acceptability of SSE among older adults with relatively mild long-term care levels who use daycare services. Furthermore, the reasons for non-participation among the 16 individuals who declined to participate in the intervention were not investigated. Concerns or anxieties about a new exercise program, such as SSE, may have influenced their decisions. Understanding these reasons will be important for improving the SSE program’s acceptability and participant engagement in future implementations. Finally, only 5 elementary step patterns were selected for this study. In contrast, Fränzel et al.^5)^ demonstrated that older adults admitted to acute geriatric wards (mean age: 79.4 ± 7.1 years; Mini-Mental State Examination: 27.5 ± 2.0; SPPB: 6.6 ± 2.2) could perform more physically demanding step patterns and cognitively challenging dual tasks (e.g., verbal fluency and calculation). Further research is needed to assess the feasibility and effectiveness of incorporating more complex step patterns within limited timeframes in a daycare service facility. In addition, the optimal intervention frequency should also be examined. Fränzel et al.^5)^ conducted SSE 5 days per week, and a higher frequency may be required to achieve improvements in physical function.

Moving forward, it will be necessary to tailor the SSE program flexibly to the physical and cognitive capacities of participants at each facility and to promote its establishment as a sustainable exercise program. Further detailed evaluation of the SSE program’s effectiveness and the safety of its long-term implementation is warranted, employing rigorous and appropriate research designs, such as quantitative assessments through non-randomized controlled trials or pilot randomized controlled trials.

## Conclusions

This study showed that the SSE can be safely implemented in daycare service facilities for older females with care levels of 3 or below. The high adherence and positive psychological and physical responses suggest that SSE is a feasible exercise program that can be used during recreational activities.
